# Machiavellian strategist or cultural learner? Mentalizing and learning over development in a resource-sharing game

**DOI:** 10.1017/ehs.2021.11

**Published:** 2021-03-10

**Authors:** Adam Baimel, Myriam Juda, Susan Birch, Joseph Henrich

**Affiliations:** 1 Department of Psychology, Health and Professional Development, Oxford Brookes University, Oxford, UK; 2 Department of Psychology, Simon Fraser University, Vancouver, British Columbia, Canada; 3 Department of Psychiatry, University of British Columbia, Vancouver, British Columbia, Canada; 4 Department of Psychology, University of British Columbia, Vancouver, British Columbia, Canada; 5 Department of Human Evolutionary Biology, Harvard University, Cambridge, MA, USA

**Keywords:** Cultural Brain Hypothesis, Social Brain Hypothesis, mentalizing, developmental, cultural learning

## Abstract

Theorists have sought to identify the key selection pressures that drove the evolution of our species’ cognitive abilities, life histories and cooperative inclinations. Focusing on two leading theories, each capable of accounting for many of the rapid changes in our lineage, we present a simple experiment designed to assess the explanatory power of both the Machiavellian Intelligence and the Cultural Brain/Intelligence Hypotheses. Children (aged 3–7 years) observed a novel social interaction that provided them with behavioural information that could either be used to outmanoeuvre a partner in subsequent interactions or for cultural learning. The results show that, even after four rounds of repeated interaction and sometimes lower pay-offs, children continued to rely on copying the observed behaviour instead of harnessing the available social information to strategically extract pay-offs (stickers) from their partners. Analyses further reveal that superior mentalizing abilities are associated with more targeted cultural learning – the selective copying of fewer irrelevant actions – while superior generalized cognitive abilities are associated with greater imitation of irrelevant actions. Neither mentalizing capacities nor more general measures of cognition explain children's ability to strategically use social information to maximize pay-offs. These results provide developmental evidence favouring the Cultural Brain/Intelligence Hypothesis over the Machiavellian Intelligence Hypothesis.

## Graphical abstract

**Figure:**
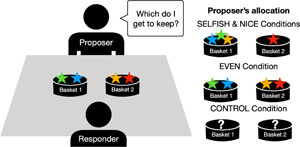

**Social media summary:** Developmental evidence favours the Cultural Intelligence Hypothesis over the Machiavellian Intelligence Hypothesis.

## 1. Introduction

What are the origins and nature of human sociality and social psychology, and how can we explain this from an evolutionary perspective? Unravelling this puzzle is challenging because evidence from paleontology, archaeology and genetics suggests that our lineage has transformed substantially over the last few million years, including a roughly 3-fold increase in brain size (Bailey & Geary, 2009; Schoenemann, 2006), greater reliance on tools and a substantial shift in our life history with the emergence of middle childhood and a long post-reproductive period prior to senescence (Boyd & Silk, 2012). Accompanying these rapid changes were energetically costly modifications to the female pelvis, permitting the birthing of large-headed infants, and a reduction in the length of gestation that made human births relatively premature from the perspective of other primates (Boyd & Silk, 2012). Because of the speed, magnitude and fitness costs of these genetic changes, current theorizing focuses on identifying the ‘autocatalytic’ or ‘runaway’ evolutionary processes responsible. Focusing on the two primary hypotheses capable of producing the requisite autocatalytic evolutionary dynamics, the Machiavellian Intelligence Hypothesis (Byrne & Whiten, 1988; Whiten & Byrne, 1997) and the Cumulative Cultural Brain Hypothesis or Cultural Intelligence Hypothesis (hereafter collectively labelled the ‘Cultural Hypothesis’), we tested the psychological implications of these two theories among 3- to 7-year-old children. Our results support the Cultural Hypothesis (Whiten & van Schaik, 2007; Muthukrishna & Henrich, 2016; Henrich, 2015b; Laland, 2017; Boyd, 2018; Gavrilets and Vose, 2006; van Schaik and Burkart, 2011), which proposes that cultural evolution generated an ever-increasing body of adaptive learned information that created selection pressures for bigger brains that were better equipped for cultural transmission. Meanwhile, we find little support for the formally modelled version of the Machiavellian Intelligence Hypothesis (McNally and Jackson, 2013), which when applied to humans proposes that our cognitive abilities, along with other anatomical changes, were driven by an arms race in strategic social reasoning.

## 2. Theoretical framework

How can we account for our species’ social psychology, sophisticated cognitive abilities, including our hypertrophied capacity for mentalizing, and our unique patterns of life history (Henrich, 2015b)? Although many ideas have been proposed, few can account for the rapid and concordant changes in our species’ brains, cognition and life history. Here, we focus on two versions of the Social Brain Hypothesis (Humphrey, 1976) that are capable of producing the requisite autocatalytic evolutionary dynamics and have been clearly laid out in formal evolutionary models. Both approaches aim to explain the variation in the cognitive abilities and computational processing power (captured by neuron number or more crudely by brain size) among primates in general (Herculano-Houzel, 2019). Crucially, both approaches can be extended to supply the evolutionary feedback dynamics necessary to explain the rapid expansion of brains and cognitive abilities in the human lineage. The leading version of the Social Brain Hypothesis, which we label the Machiavellian Intelligence Hypothesis (for clarity), proposes that big brains and sophisticated cognitive abilities result from the selection pressures for strategic thinking applied to managing relationships in larger, or more intensely social, groups (Byrne & Whiten, 1988; Whiten & Byrne, 1997; Dunbar, 1998). Favoured individuals, in this view, are better able to track and strategically deploy information, including about third parties, regarding the strategies or choices of others. These psychological abilities allow them to better trick, manipulate and deceive others, as well as to sustain longer-term alliances or partnerships. In this view, the complexity of primate social life is driven by some external pressure, like predation (Dunbar & Shultz, 2007). For the human case, the required runaway dynamics arise from an ever-escalating social competition in strategic thinking in which selection favours competitors who can reason one step farther than others – as in the backward or forward induction required of agents in standard game theory (Trivers, 1971; Binmore, 1991). One potential reason why this runaway social competition occurred in the human lineages, but not in other species, may be due to intergroup competition, where members of the same species became potentially dangerous predators (Bailey & Geary, 2009). To facilitate the application of strategic reasoning in social interactions – game-theoretic thinking – this view holds that humans have evolved greater abilities to represent others’ mental states (i.e. their beliefs and preferences) – mentalizing – and employ these abilities to exploit or manipulate conspecifics (e.g. Byrne & Whiten, 1991).We note that Byrne and Whiten's (1988) original conceptualization of the Machiavellian Intelligence Hypothesis was broader than what we have presented here including, for example, primates’ skills in managing coalitions; we focus more narrowly on the part of the hypothesis capable of generating the necessary runaway dynamics.

In contrast, the Cultural Hypothesis proposes that combinations of individual and social learning generate a pool of adaptive non-genetic information, which may take many forms including foraging skills, food preferences, tool-using techniques, communicative signals, ally preferences or socially strategic tactics (Muthukrishna & Henrich, 2016; Whiten & van Schaik, 2007; Gavrilets & Vose, 2006; Reader et al., 2011; Henrich, 2015b; Street et al., 2017; Laland, 2017; van Schaik & Burkart, 2011).We note that, in this context, the Cultural Hypothesis converges with the Embodied Capital Hypothesis (Kaplan et al., 2000). We focus on the former because those working under this rubric have explicitly analysed the role of cumulative cultural evolution. Note that, while Gavrilets and Vose (2006) is presented under the rubric of the Machiavellian Intelligence Hypothesis, we feel that the dynamic processes embedded in the model actually capture a version of the Cumulative Cultural Brain Hypothesis. The emergence of this pool of adaptive information creates selection pressures favouring brains that are better able to acquire, store, organize and re-transmit this body of fitness-enhancing information. Applied to the human lineage, our ancestors crossed a theoretical threshold in which adaptive know-how and preferences could substantially accumulate and accelerate over generations. This further increased the selection pressure for brains that were better able to acquire, store, organize and re-transmit this information. The better at cultural learning human ancestors became, the more rapidly cultural evolution could accumulate large pools of adaptive know-how, and the greater the selection pressures became on genes for building brains that were better able to tap into this distributed information. Here, mentalizing evolved in order to improve cultural learning, to better extract knowledge, motivations, beliefs, intentions and strategies, from other’ minds. Moreover, mentalizing capacities for making inferences about others knowledge states also probably supported teaching, communicative cuing and pedagogy (Hoehl et al., 2014; Kline, 2015; Skerry et al., 2013; Ho et al., 2017; Csibra and Gergely, 2011). By this account, better mentalizers should be more selective in their learning, and more attuned to accurate copying; for example, good mentalizers should be better able to copy intentional over incidental or accidental behaviours. Indeed, while non-selective imitation (‘overimitation’) can be a good cultural learning strategy that ensures that all key behaviours are copied (e.g. Chudek et al., 2016; Hoehl et al., 2019) – learning may be made more efficient by selectively distinguishing the necessary from the irrelevant. Superior mentalizing may equip learners with the capacity to be more selective, in part as it helps learners figure out what is necessary and what is not (e.g. Brosseau-Liard et al., 2015).

The distinct psychological implications of these two theories are important because they otherwise make similar predictions about other relationships, such as those between sociality, computational power (neuron number; Herculano-Houzel, 2019) and breeding patterns (Muthukrishna et al., 2018; Dunbar & Shultz, 2007; McNally et al., 2012; McNally & Jackson, 2013; Fox et al., 2017; Street et al., 2017). Although both approaches do emphasize the importance of our species’ mentalizing or ‘mind-reading’ abilities, they propose that these mentalizing abilities will be put into primary service in quite different ways. Specifically, the Machiavellian Intelligence Hypothesis holds that humans readily develop the ability and motivation to out-smart others, by out-mentalizing them, especially in competition for desirable resources. In contrast, the Cultural Hypothesis proposes that mentalizing abilities will first develop for, and be deployed most commonly, in the service of cultural learning, not primarily for strategically out-witting others. The empirical question is: do children *initially* put their mental abilities to work in learning from others or to exploiting their opponents for personal gain? If either or both of these selection pressures were key drivers in human evolution, we should be able to detect them in contemporary human cognition and decision-making (Bjorklund & Pellegrini, 2000; Barrett et al., 2014).

Taking advantage of the gradual development of prosociality (House et al., 2013), mentalizing (Birch et al., 2016) and norm adherence in children (House et al., 2019; Amir & McAuliffe, 2020), we allowed the psychological hypotheses to compete in a simple experimental design administered to 280 children (51% female) ranging in age from 3 to 7 years old in Vancouver, Canada. Our approach was two pronged. First, we observed children's decisions in a resource distribution game in which they could win stickers – a valued resource. Importantly, the particulars of the game and its conditions were designed such that children's decisions could reflect either the outcomes of imitative cultural learning or strategic social reasoning. Second, we assessed children's capacities for mentalizing in three ways: using (1) a classic false-belief task (*N* = 276; Wimmer and Perner, 1983); (2) a storybook instrument (*N* = 100; Blijd-Hoogewys et al., 2008); and (3) parental reports (*N* = 150; Tahiroglu et al., 2014). Critically, the latter two measures operationalize mentalizing as a suite of related multidimensional capacities implicated in reasoning about others’ mental states, allowing us to triangulate children's developing capacities more broadly then what is captured by common ‘Theory of Mind’ measures like the binary outcome of the false-belief task (Schaafsma et al., 2015). In a subset of the sample (*N* = 118), we also measured children's general cognitive abilities (McGrew & Woodcock, 2001). In this experimental design, the Cultural Hypothesis predicts that mentalizing should be associated with the cultural acquisition of relevant and intentional actions, preferences or strategies while the Machiavellian Intelligence Hypothesis predicts that mentalizing will be associated with behaviour that maximizes pay-offs by taking advantage of social information in a zero-sum interaction. Our measure of general cognitive abilities provides a valuable control.

Of course, people in real life engage in both cultural learning and Machiavellian strategizing, and rely on mentalizing in both forms of social interaction. At its core, the Cultural Hypothesis proposes that human social life is constructed by an array of culturally transmitted social norms that generate both reputational consequences and signalling opportunities. As a result, the first thing an individual must do to survive and thrive in this world is deploy their cultural learning to figure out the local norms. Only then, having acquired the local norms, can they begin to exploit and manipulate at the edges. In contrast, under the Machiavellian Intelligence Hypothesis, the need to first learn social norms before engaging in strategic behaviour plays no role. This suggests that cultural learning will play little role in strategic decision-making. Thus, the design of our experiment allows for an examination of the ways in which children employ their developing capacities for mentalizing: do they, when faced with a zero-sum social decision, deploy these cognitive abilities in the service of cultural learning or strategic reasoning (or both)?

Understanding the ontogeny of any phenotype has stood at the core of evolutionary approaches at least since Darwin (1959), and was canonized by Tinbergen (1963) in his ‘Four Questions’. Here, we study children during a developmental period when they are known to internalize social norms (House et al., 2013, 2019), increase their general cognitive skills (McGrew & Woodcock, 2001) and sharpen their mentalizing abilities (Wellman & Liu, 2004). We note that there remain, however, active debate as to when precisely children (or even infants) become able to make inferences about the mental states of others (Baillargeon et al., 2010; Tomasello, 2018; Poulin-Dubois et al., 2018). That said, much of the literature on the development of mentalizing has long focused on children in this age range and some comparative evidence suggests that by this age children's social cognition is more sophisticated than that of some of our closest primate relatives. This provides us with important empirical opportunities that are unavailable with adult participants. Our data provide evidence for the early development of these behaviours and abilities. We might have observed, for example, that imitation develops only slowly over this period but that even young children were quite inclined to make equal allocations. We do not find this. Or, we might have found that while young children rely on imitation, older children became fierce Machiavellians. We do not find this either. Instead, we find that young children are powerful imitators but possess only weak inclinations toward equitable offers, which increase slowly over this period. For an overview of the importance of studying child development for evolutionary approaches to humans see Barrett (2014), Bjorklund and Pellegrini (2000) and Henrich and Muthukrishna (2021).

## 3. Methods

### 3.1. Participants

In the greater Vancouver area (Canada), 280 children (136 males; 144 females; 2 with sex unreported) aged from 2.91 to 6.93 years (mean = 4.48, SD = 0.94) were recruited to participate in this study from 22 daycare centres (*N* = 201), a local science museum (*N* = 55) and the child subject pool at the University of British Columbia (*N* = 24). The family income of participating children ranged from 20,000 to 220,000 CAD at our different sampling sites around the city (median = 100,000 CAD). The median family income in the greater Vancouver area in 2015 was around 72,000 CAD (Statistics Canada, 2017). Most of the children had one (*N* = 234) or two siblings (*N* = 27).This study was approved by the University of British Columbia Behavioural Research Ethics Board. Written informed consent was obtained by the parents of participating children in addition to children's verbal assent to participate, and children were given the option to withdraw at any point during the study.

### 3.2. Materials and procedures

Participants completed a battery of assessments: (a) the sticker bargaining game; (b) a false-belief test (Wimmer & Perner, 1983); (3) the Theory of Mind (ToM) storybooks (Blijd-Hoogewys et al., 2008); and (4) a test of general cognitive abilities (McGrew & Woodcock, 2001). Participating children recruited from daycares completed the assessments in a round-robin style with different research assistants making one to three visits per daycare centre. Parents or guardians of participating children provided demographic information and filled out an observation instrument on their child's mentalizing capacities (Tahiroglu et al., 2014). The parent/guardian questionnaire was completed either as the child participated in the other tasks (at the Science Museum and in-lab) or was sent home with participating children at daycare centres and collected at a later time. As some of the assessments (e.g. the ToM storybooks and the cognitive ability tests) required lengthy and/or returning sessions with the children, we do not have complete data for all participating children. This is primarily due to children's absence on returning visits to the daycare centres and take-home questionnaires not being returned.

#### 3.2.1. The sticker bargaining game

**Figure 1.**
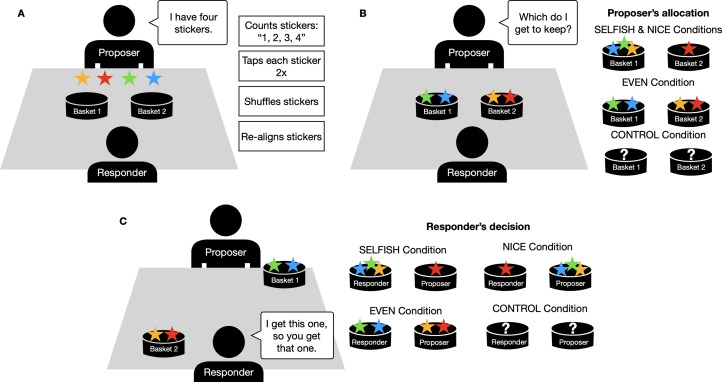
Phases of the sticker game. (a) Initial set up and irrelevant behaviours, (b) proposer's allocations and how they differed between conditions and (c) responder's decision and how they differed between conditions. The participating child was seated at the table and observed two adult models play three rounds of the game before taking the place of the proposer and playing against the same responder they had just observed. In the Control condition, a box was placed over the baskets before the Proposer allocated the stickers and taken away after the Responder had decided which basket to take and which to give back to the Responder – leaving participants unaware of the decisions made in the game.

The sticker game involved two active players, a proposer and a responder (see Figure 1). The proposer had to decide how to allocate four stickers between two baskets; the responder then had to pick which basket they wanted, which left the proposer with the remaining basket. When responders are assumed to prefer more stickers to fewer stickers, game theory predicts that sticker-maximizing proposers will make a 2–2 division between the baskets. Procedurally, children first watched a live demonstration of the game in which two adult models interacted for three rounds, ostensibly as an instructional aid. A third adult experimenter laid out the the stickers in front of the proposer at the beginning of each round. In all demonstrations, the proposer initiated each round by performing five actions: announcing that they had four stickers (‘I have four stickers’), counting them out loud (‘One, two, three, four’), tapping on each sticker twice with a finger, shuffling them around into a different order and realigning them into a straight line. The proposer then allocated the stickers to the baskets, and asked the responder, ‘Which do I get to keep?’ – prompting the responder's decision. Importantly, the demonstration varied (a) the proposer's allocations, (b) the responder's preferences and (c) whether or not the participants could actually observe any of the allocations or sticker pay-offs.

Children were randomly assigned to one of four conditions, labeled Control, Even, Selfish, Nice (Table 1), and played for four rounds in the role of proposer against the *same person they had just observed in the responder role in the demonstration*. Table 1 summarizes these treatments and predictions:

1. *Control condition*: the proposer in the live demonstration was given the four stickers, they then performed the five actions described above. After stating that they were to put the stickers in the baskets, and *before* asking which they got to keep, the experimenter in the demonstration placed an occluding box over the baskets that had one side cut out such that the adult proposer and the responder could see the baskets and the placement of the stickers but the participant could not. The responder selected one of the baskets from within the box, so participants also could not see how many stickers either player retrieved. The box was removed and then replaced prior to the proposer's allocations in the following rounds. This treatment provides a comparative baseline for how children will allocate stickers at test in the absence of information about the proposer's or the responder's preferences. In the other conditions, children had full view of all decisions and outcomes.
2. *Even condition*: the proposer split the stickers evenly, leaving the responder with no choice but to return two stickers and keep two for themselves (basket choice was counterbalanced across the sample). Since this 2–2 split provides no additional information as to the responder's preferences, Machiavellians who should adjust their strategies in light of the responders’ behaviours are predicted to act as they would if they had been in the Control (where they have similarly no additional information as to the responder's preferences). Cultural learners, however, should tend to copy the model and split the stickers evenly.
3. *Nice condition*: The proposer distributed the stickers unevenly with three stickers in one basket and one in the other (the order of which was counterbalanced across participants). The responder was then ‘nice’ and always picked the basket with only one sticker. Here, both good cultural learners and Machiavellians should allocate unevenly, with cultural learners copying the model and Machiavellians adjusting to best exploit their opponent.
4. *Selfish condition*: the proposer allocated the stickers unevenly but now the responder was ‘selfish’ and always took the basket with three stickers. Here, cultural learners should copy the uneven allocation tendencies of the model, while good Machiavellians should recognize the sticker-maximizing tendencies of their opponent and pick an even 2–2 allocation.

**Table 1.** Sticker game decision matrix and by condition predictions

Conditions	Proposer's actions	Responder's actions	Cultural learners’ divisions	Machiavellian divisions
Control	Unseen	Unseen	Baseline	Baseline
Even	Allocates 2–2	Picks 2 sticker cup	Even (2–2)	Baseline
Nice	Allocates 3–1	Picks 1 sticker cup	Uneven (3–1)	Uneven split (4–0, 3–1)
Selfish	Allocates 3–1	Picks 3 sticker cup	Uneven (3–1)	Even split (2–2)

After the demonstrations, children were asked if they wanted to play and were placed into the role of the proposer, playing against the *same responder* that they had just seen in the demonstration. When the child first took the place of the proposer, they were asked whether they liked stickers (in general). Four participants said they did not. At the outset of each round of the testing phase, children were asked whether or not they liked the specific stickers that had been laid out in front of them by the experimenter. To these queries, 17 children indicated that they did not like the *specific* stickers on that round. Preliminary analyses revealed no robust relationship between children's report of liking of the stickers and their choices, so we did not exclude children based on their reported sticker preferences. Participants played the game for four rounds with the responder playing the same strategy that the child saw in the demonstration. In the Control condition, however, the responder's behaviours at test were dependent on the child's allocations but were pre-determined and counterbalanced across the sample. If the child distributed evenly, the responder always chose either the left or right basket. If allocations were uneven, the responder was randomly assigned *a priori* to be either nice (*N* = 12) or selfish (*N* = 14). This was also the case in response to uneven allocations in the Even condition (nice, *N* = 10; selfish, *N* = 4). After each round, the stickers that the children obtained in the game were placed in a small plastic bag for them to take home.

#### 3.2.2. Measures of mentalizing

We measured children's mentalizing abilities in three ways:

1. *The Sally Anne Task* (Wimmer & Perner, 1983): in this task children were presented with a live demonstration of a false belief test using hand puppets in a ‘change of location paradigm’. The test involved two characters, ‘Sally’ who had a basket and ‘Anne’ who had a box. The test began with Sally placing a toy in her basket. Sally then left the scene to ‘go play outside’. While Sally was away and could not see what took place, Anne took the toy out of Sally's basket to put it into her own box. Sally then returned and the child participant is asked three questions: ‘Where is the toy now?’; one memory question, ‘Where was the toy at the beginning?’; and the focal belief question, ‘Where will Sally look for her toy when she comes back in from playing outside?’ Children are said to pass the test when they reply that Sally will look for the toy inside her own basket (1 = pass; 0 = fail) – that is, where she had left it (and *not* where the child knows it to currently be). As is standard practice to insure that participants understood where Sally had actually placed the toy, and where it was in reality after it was moved by Anne, the experimenter corrected the participant if they had responded incorrectly to either of these two questions before asking the focal test question. Incorrect responses on the false belief item were not corrected.
2. *The ToM storybooks* (Blijd-Hoogewys et al., 2008): this instrument consists of six storybooks portraying a protagonist, Sam, who experiences various emotions, desires and thoughts in a series of brief stories about this character, his friends and his family. The storybooks, which were read aloud by an adult experimenter, consist of 34 tasks, with assessments of five components of mentalizing: (a) emotion recognition; (b) distinguishing between physical and mental entities; (c) understanding that seeing leads to knowing; (d) prediction of behaviours and emotions from desires; and (e) prediction of behaviours and emotions from beliefs. An overall ‘Theory of Mind’ score is indexed by the sum-total of coded responses, ranging from 0 to 110 on the basis of a continuous scoring system. The task takes 40–50 min to complete. As an instrument of various aspects of mentalizing, these storybooks have been shown to have robust internal consistency, test–retest reliability, inter-rater reliability, construct validity and convergent validity.
3. *The Children's Social Understanding Scale* (CSUS-short form; Tahiroglu et al., 2014): parents of a subset of our sample also completed an 18-item parent-report questionnaire of their child's mentalizing capacities. The CSUS asks parents to reflect on their child's capacities for reasoning about mental states such as beliefs (e.g. ‘My child understands that telling lies can mislead other people’), knowledge (e.g. ‘My child uses words that express uncertainty’), perception (e.g. ‘My child thinks that you can still see an object even if you're looking in the opposite direction’. *reverse-coded*), desires (e.g. ‘My child talks about what people like or want’), intentions (e.g. ‘My child talks about the difference between intentions and outcomes’) and emotions (e.g. ‘My child talks about conflicting emotions’). The 18-item scale is reported to have good psychometric properties, and has been validated in samples of children aged 3–8 years of age.

#### 3.2.3. General cognitive abilities

To assess children's general cognitive abilities, a subset of our sample completed the Brief Intellectual Ability test (BIA; McGrew & Woodcock, 2001). The BIA was designed to assess cognitive abilities in children older than 2 years. An overall score is derived from the outcomes of three cognitive tests involving verbal comprehension, concept formation and visual matching that assessed verbal skills, fluid reasoning and processing speed. For our analyses, scores on the test were age-normalized using the scoring programme provided by the test creators.

### 3.3. Sticker game response coding

Children's behaviours provided us with a rich set of data. We first coded children's sticker allocations and tracked their relative frequency across rounds and conditions to assess the extent of imitative cultural learning. Then, we also coded allocations as to whether they reflected pay-off-maximizing choices (game theory). This permits us to estimate the contributions of mentalizing and general cognitive abilities to both imitation of the model proposer and strategic exploitation of the responder. Lastly, we counted if and how many of the proposer's seemingly irrelevant behaviours – as seen in the demonstration (e.g. counting, tapping, shuffling) – the child reproduced on each test round to provide a measure of overimitation.

## 4. Results

We analysed our data in two steps. First, we considered how well the data fit the predictions arising from the Cultural Hypothesis by asking if, and how much, children tended to imitate the allocations and behaviours of their model/demonstrator. Second, we contrasted this analysis with how well children's behaviour fit the predictions derived from the Machiavellian Intelligence Hypothesis. Crucially, this approach to analysing our data allows for the possibility that we could find mixed evidence, with the data supporting both sets of predictions and theories.

### 4.1. Are children cultural learners in this zero-sum situation?

To assess the impact of our four treatments (*t*), we began by coding children's allocations into a binary variable, as either even splits (2/2, so *d*_*i*,*j*,*s*_ = 1) or uneven (i.e. 3 and 1 or 4 and 0, so *d*_*i*,*j*,*s*_ = 0). The variable *i* indexes the round, *j*indexes the individual and *s* marks the sampling site. We modelled these decisions in a series of logistic regressions. To account for the non-independence of repeated responses across rounds and data collection in different sites, we adjusted all standard errors by clustering both within subjects and within sampling sites (22 daycares, science museum or in-lab). We estimate the regression equation (1) below. The coefficient on condition, *C*_*t*_, captures the effects of our four treatments, using our Control condition as the reference. The coefficient on round, *R*_*i*_, reveals the average effect of personal experience per round of repeated play. The coefficient *β*_*t*_ captures the effect of the interaction of treatment and round, which is crucial since we expect individual learning to have different effects in different treatments. The coefficient on children's ages, *A*_*j*_, allows us to examine how children's inclination to offer even splits develops from age 3 to 8 years in this population. The coefficient *M*_*j*_ controls for the reported sex of our participants (*sex* = 1 is male; which was centred to ease interpretation of our focal predictors for the whole sample).1
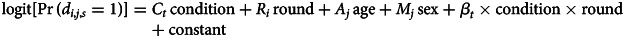


**Figure 2.**
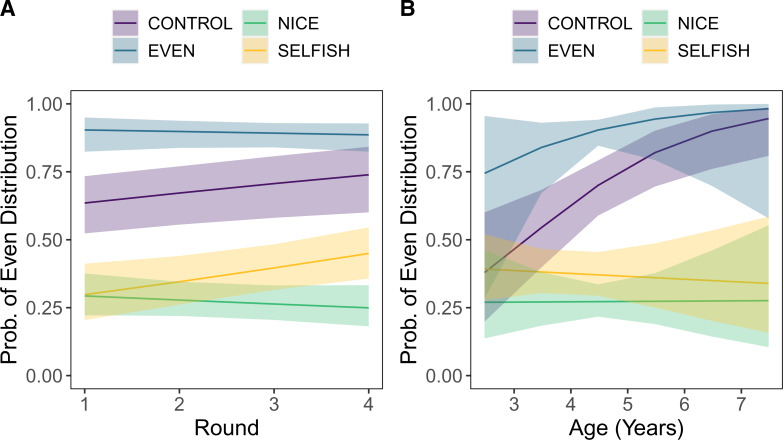
Predicted probability of even distributions in each condition across the four rounds (left panel) and age (right panel). Predictions for panels (a) and (b) were generated from models 4 and 5, respectively, in Table 2. The shaded regions show the 95% confidence intervals based on two-way clustering.

Figure 2 illustrates our key results and Table 2 provides greater detail. For each of our conditions, the left panel reveals the predicted probabilities of even allocations across the four rounds of play (as estimated by model 4 in Table 2). The right panel shows the age trajectories in allocation strategies for each condition (as estimated by model 5 in Table 2). The most striking result is the tendency of children to imitate the proposer they observed in the demonstration. Relative to the Control condition, children who saw an even distribution were much more likely to distribute their stickers evenly (the blue line at the top of both plots). In round 1, for example, the percentage of equal allocations increased from 62% in the Control condition to 91% in the Even condition. Similarly, when children saw a proposer divide the stickers unevenly in either the Selfish or Nice conditions, they allocated their stickers much less evenly at test. In round 1, the percentage of even allocations dropped to 28% in both the Nice and the Selfish conditions. This is 34% below the frequency of equal splits observed in the Control condition. Table 2 shows that, even holding participants’ age, sex and round of play constant, those who observed an even split were substantially more likely to offer an even split while those who observed uneven splits were substantially less likely to propose an equal division.

**Table 2.** Logistic regression models to predict uneven vs. even sticker allocations

	Sticker allocations (0 = uneven; 1 = even)				
	Model 1	Model 2	Model 3	Model 4	Model 5
	OR (95% CI)	OR (95% CI)	OR (95% CI)	OR (95% CI)	OR (95% CI)
Intercept	2.22***	1.97**	1.95***	1.74**	2.05***
	(1.35, 3.63)	(1.12, 3.45)	(1.18, 3.20)	(1.09, 2.76)	(1.29, 3.26)
Even condition	3.69***	3.69***	3.88***	5.41***	4.02***
	(1.69, 8.08)	(2.01, 6.80)	(2.13, 7.08)	(2.58, 11.34)	(2.14, 7.53)
Nice condition	0.17***	0.17***	0.17***	0.24***	0.16***
	(0.09, 0.34)	(0.09, 0.32)	(0.09, 0.32)	(0.13, 0.43)	(0.09, 0.30)
Selfish condition	0.26***	0.26***	0.27***	0.24***	0.25***
	(0.14, 0.50)	(0.12, 0.56)	(0.13, 0.55)	(0.11, 0.52)	(0.13, 0.49)
Round (0 = round 1)		1.09**	1.09**	1.18**	1.09**
		(1.01, 1.17)	(1.00, 1.17)	(1.04, 1.33)	(1.00, 1.18)
Age (years, centred)			1.27	1.27	1.96***
			(0.94, 1.72)	(0.94, 1.73)	(1.28, 2.98)
Sex (0 = proportion of males)			1.52*	1.52*	1.49*
			(0.98, 2.35)	(0.98, 2.37)	(0.96, 2.31)
Even condition × round				0.80*	
				(0.61, 1.04)	
Nice condition × round				0.79***	
				(0.66, 0.94)	
Selfish condition × round				1.06	
				(0.83, 1.35)	
Even condition × age					0.92
					(0.30, 2.77)
Nice condition × age					0.51**
					(0.30, 0.89)
Selfish condition × age					0.49***
					(0.32, 0.74)
Observations	1080				
Participants	273				
Sites	24				

*Notes:* Coefficients are presented as odds ratios, so ‘1’ indicates no effect. Standard errors and confidence intervals are robust and use two-way clustering on both individuals and sites. The 95% confidence intervals are reported below each coefficient in parentheses. The Control condition (intercept; controlling for other variables) is the reference category for condition effects. Round of the game was treated as a continuous variable, and thus condition by round interactions represent changes across the rounds in each condition (see Figure 2a). Sex was centred on the percentage of males to ease interpretation of the other coefficients for the entire sample. Age (years) was mean-centred, and developmental trajectories in each condition estimated by model 5 are plotted in Figure 2b. For those interested in significance testing, ***, ** and * indicate *p*-values below 0.01, 0.05 and 0.1, respectively.

Unlike the impact of cultural learning illustrated above, individual learning played little role over the four rounds of repeated play (Figure 2, left panel). In three of our conditions (not Even), children altered their allocations in ways that increased their pay-offs – see the coefficients in model 4 (Table 2) for round (in Control) and the interactions of each condition and round. However, these effects are small and not always estimated with precision. The Even condition (interacted with round) appears slightly anomalous but this results from the fact that nearly all children in this condition made even allocations in the first round. Overall, the impact of cultural learning from the demonstrator dominates individual experience, even in the last round.

As children got older, Figure 2 (right panel) reveals how their responses varied across our conditions (model 5 in Table 2). In the Control condition, older children were about twice as likely to offer an equal split for each additional year, a pattern consistent with much existing developmental research (Blake et al., 2015; Henrich & Muthukrishna, 2021). In contrast, when they first observed a model offer an uneven split, they became much less likely to make an even split (compared with baseline) as they got older. That is, the imitative cues vastly dominated any impact of enculturation on making equal offers observed in the Control condition, indicating that older children were more affected by the actions of the demonstrator. Finally, observing an equal allocation prior to playing had little impact as children aged (as baseline responses in the Control converged with behaviours in the Even condition).

To verify these results, we conducted a supplemental study with 39 additional participants that sought to (a) replicate our main finding for the Even and Selfish conditions and (b) probe children's understanding of the task. The results, detailed in Section S3 in the Supporting Information, replicate the relevant findings just discussed and reveal how children understood the rules of the game.

#### 4.1.1. Do mentalizing abilities improve cultural learning?

To further test predictions from the Cultural Hypothesis, we analysed the relationship between *selective* imitation in the sticker game and our three measures of mentalizing, controlling for general cognitive ability (BIA). If mentalizing is *for* sharpening the accuracy and targeting of cultural learning, then we would expect that better mentalizers would copy fewer of the demonstrators’ irrelevant actions (e.g. tapping, counting, shuffling). Recall that, before distributing the stickers in each round, the experimenter consistently performed five actions that were not connected to the actual sticker allocations. At test, we tallied how many of these behaviours children imitated, and modelled the total counts in each round in a series of Poisson regressions (Table 3), again using robust standard errors adjusted by clustering on both subjects and sampling site.

**Table 3.** Poisson regression models to predict counts of overimitation from mentalizing and cognitive ability

	Overimitation (counts of irrelevant actions by round)		
	False belief	ToM storybooks	CSUS
	OR (95% CI)	OR (95% CI)	OR (95% CI)
Intercept	0.41***	0.30***	0.34***
	(0.30, 0.56)	(0.21, 0.42)	(0.22, 0.53)
Mentalizing	0.67**	0.9**	0.49
	(0.46, 0.97)	(0.95, 1.00)	(0.14, 1.74)
Cognitive ability (centred)	1.05**	1.04***	1.04***
	(1.01, 1.09)	(1.02, 1.07)	(1.02, 1.07)
Mentalizing × cognitive ability	0.98	1.00	0.96
	(0.94, 1.02)	(0.99, 1.01)	(0.87, 1.07)
Observations	463	300	272
Participants	116	75	68
Sites	18	17	17

*Notes:* Coefficients are presented as odds ratios (OR), so ‘1’ indicates no effect. Standard errors and confidence intervals (CI) are robust and use two-way clustering on both individuals and sites. The 95% confidence intervals are reported below each coefficient in parentheses. False belief was coded as pass/fail (1/0). Theory of Mind (ToM) storybook and Children's Social Understanding Scale (CSUS) scores were centred. Models with additional controls are presented in the Supporting Information: false belief (Table S1), ToM storybooks (Table S2) and CSUS (Table S3). For those interested in significance testing, ***, ** and * indicate *p*-values below 0.01, 0.05 and 0.1, respectively.

**Figure 3.**
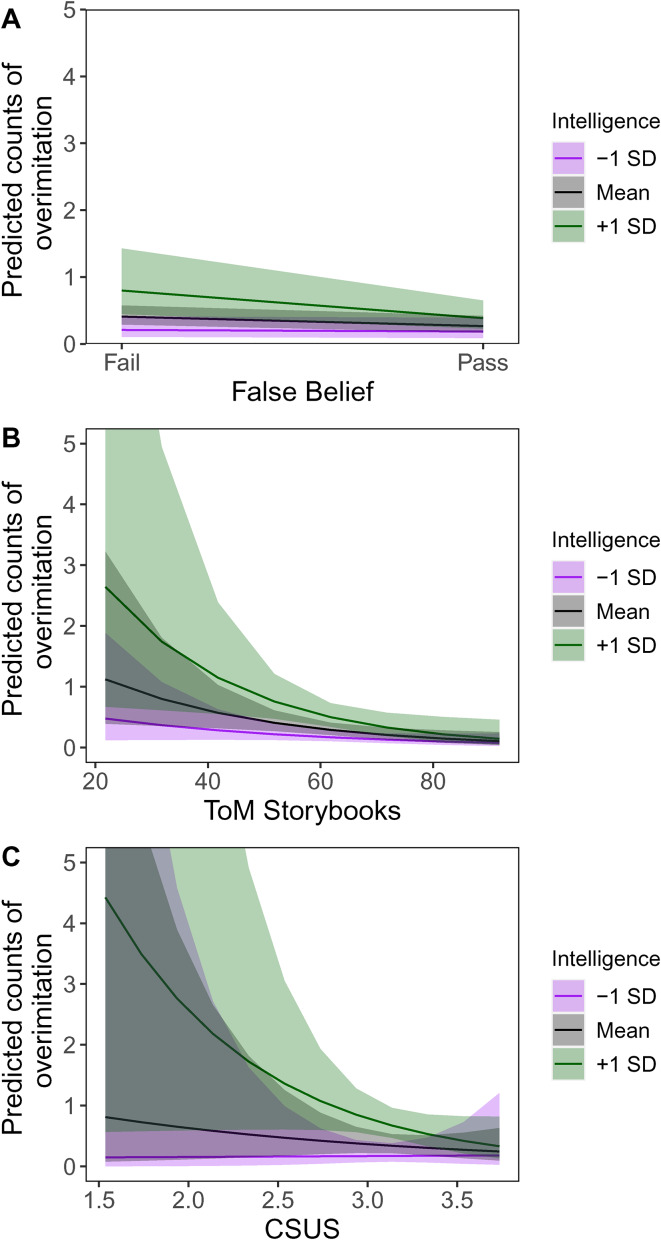
Predicted overimitation counts by mentalizing and general cogntive ability scores. Shaded regions are 95% confidence intervals. Predictions were generated from models presented in Table 3.

Across models, we observed clear associations between children's cognitive abilities, mentalizing capacities and the extent of their overimitation. Figure 3 illustrates that greater mentalizing capacities, as indexed by (a) passing the false-belief test (Table S1), (b) higher scores on the ToM storybooks (Table S2) or (c) greater parent-reported capacities for reasoning about mental states (CSUS; Table S3), were associated with decreased overimitation (summarized in Table 3). As shown in the figure, the effects of mentalizing are large, although the point estimate for the coefficient on the CSUS – the largest effect – is estimated with great uncertainty. In some of our supplemental analyses (Tables S3 and S4), the coefficients on CSUS are estimated with much greater precision, although this depends on the specification. In contrast to mentalizing, greater cognitive abilities as measured by the BIA are associated with *more* overimitation. Indeed, the data hint that the stronger the cognitive performance of children on the BIA, the greater the impact of mentalizing on overimitation.

One interpretation of these results is that many or most children are motivated to overimitate, but remain limited by their cognitive abilities in accomplishing this. Children with stronger cognitive abilities, as captured by the BIA, are able to overmitate more. Notably, detailed analyses indicate that no one of the three subscales on the BIA is driving the observed relationship with overimitation (Table S4). This work suggests that it is mentalizing abilities, not these more general-purpose cognitive abilities, that make children more effective and accurate cultural learners.

### 4.2. Are children good Machiavellians in this bargaining context?

To test the focal predictions of the Machiavellian Intelligence Hypothesis, we estimated the contributions of mentalizing and cognitive abilities on children's capacities to exploit the responder in order to maximize their own sticker pay-offs. The Pay-off Maximizing Choice – the PMC – varied by condition such that ‘even’ allocations were pay-off maximizing in the Even and Selfish conditions while ‘uneven’ distributions were pay-off maximizing in the Nice condition. In the Control condition, participants were blind to the responder's strategy in the demonstration, and thus ‘even’ allocations were coded as pay-off maximizing until the participant distributed stickers unevenly, which would reveal the responder's selfish or nice strategy (*a priori* counterbalanced). If selfish, then ‘even’ distributions on the *following* round were coded as pay-off maximizing. If the responder was nice, then ‘uneven’ distributions on the *following* rounds was coded as pay-off maximizing. Children's allocations, indexed as being either pay-off-maximizing (PMC = 1) or not (PMC = 0) were modelled in a series of logistic regressions with standard errors adjusted by clustering on subjects and sampling sites. The results that follow were robust to alternative codings of allocations in the Control condition. In additional models, we treated all uneven allocations in the Control condition as *not* pay-off maximizing, and in others treated the first uneven allocation (if the responder was ‘nice’) as pay-off maximizing despite the child probably ‘lucking into’ the higher pay-off – neither of which made any substantial changes to the estimates presented in Table 4.

**Table 4.** Logistic regression models to predict pay-off maximizing choices from mentalizing and cognitive ability

	Sticker allocations (1 = pay-off maximizing choice)		
	False belief	ToM storybooks	Parental report (CSUS)
	OR (95% CI)	OR (95% CI)	OR (95% CI)
Intercept	2.49***	2.47***	1.89***
	(1.37, 4.52)	(1.58, 3.86)	(1.23, 2.91)
Mentalizing	0.85	1.02	0.65
	(0.42, 1.69)	(0.99, 1.04)	(0.16, 2.62)
Cognitive ability (centred)	1.01	1.01	1.01
	(0.98, 1.03)	(0.97, 1.05)	(0.97, 1.05)
Observations	463	299	271
Participants	116	75	68
Sites	18	17	17

*Notes:* Coefficients are presented as odds ratios (OR), so ‘1’ indicates no effect. Standard errors and confidence intervals (CI) are robust and use two-way clustering on both individuals and sites. The 95% confidence intervals are reported below each coefficient in parentheses. False belief was coded as pass/fail (1/0). ToM storybook and CSUS scores were centred. Models with additional controls are presented in the supplemental: false belief (Table S5), ToM storybooks (Table S6) and CSUS (Table S7). For those interested in significance testing, ***, ** and * indicate *p*-values below 0.01, 0.05 and 0.1, respectively.

In contrast to our analyses of overimitation, these analyses reveal only weak and poorly estimated relationships between making pay-off-maximizing strategic choices and any of our measures of mentalizing or general cognitive abilities (Table 4; see Tables S5–S7 for models with additional controls). Two of our measures of mentalizing suggest that greater mentalizing is associated with less pay-off-maximization (the opposite of the prediction from the Machiavellian Intelligence Hypothesis) and one measure suggests a tiny positive effect of mentalizing on pay-off-maximizing choices; however, all estimates are paired with large confidence intervals that stretch across 1. Focusing on more general cognitive abilities, a child's BIA scores reveals a small positive association with pay-off-maximization, although this too is poorly estimated. These results provide no support for the idea that either a child's mentalizing skills or cognitive abilities are deployed to anticipate the predictable actions of one's partners in order to select the pay-off maximizing behaviour. That is, children seem to ignore information about their interaction partner and instead rely on their cultural model for how to behave in this context.

## 5. Discussion

In this paper, we present a simple experiment designed to examine how children trade off information relevant for cultural learning vs. information about their partner in a novel, zero-sum interaction involving real pay-offs. The experiment was designed to test a simple set of contrasting predictions stemming from what many perceive as the two leading evolutionary approaches to understanding the primary selective processes that drove our species’ genetic evolution and may explain the unique position we hold in the natural world. Of course, these are broad ranging theories that make myriad predictions about human evolution, life history, neural computational power and several features of psychology, so our efforts here, however stark, remain but one contribution to a rich and growing body of evidence from several disciplines. Nevertheless, keeping the broader theoretical frames in mind is crucial to cumulative scientific progress (Muthukrishna & Henrich, 2019).

Naturally, readers may question how we dispensed with the myriad other proposals regarding the key drivers of human evolution and our species’ immense ecological success. To begin, we emphasize that many important lines of work that might seem to be excluded actually fall under one of the two approaches we delineate. For example, approaches that emphasize teaching and pedagogy are part of the Cultural Hypothesis (Laland, 2017; Henrich, 2015a). Similarly, approaches that emphasize partner choice and alliance building can generally be incorporated under the Machiavellian rubric (Barclay, 2011). Beyond this, as noted above, we applied two criteria. First, the approach had to provde a ‘process-based’ theory that offered the requisite evolutionary dynamics capable of generating the kind of rapid transformations that appear in the fossil record and our lineage's genome. This dispenses with most alternatives. Second, we focus only on theories that had been formally modelled in some way. In our experience, many proposals fall apart when modelling is attempted because they lack sufficient clarity to be translated into mathematical terms, or, they are trivial and reduce simply to a ‘magic mutation’.

The two evolutionary hypotheses we tested are both variants of the Social Brain Hypothesis (Humphrey, 1976): the Machiavellian Intelligence Hypothesis (Byrne & Whiten, 1988; Whiten & Byrne, 1997; McNally et al., 2012; McNally & Jackson, 2013) and the Cultural Hypothesis (Muthukrishna et al., 2018; Laland, 2017). The autocatalytic version of the former suggests that the driving selection pressures in human evolution arose from an arms race in strategic thinking, with a focus on deception, manipulation, exploitation and alliance-making created by living in larger and/or more social groups. In contrast, the latter hypothesis argues for a synergy between genes and culture in which cultural evolution generates an ever expanding body of adaptive cultural information that, in turn, favours brains that are better at acquiring, storing and organizing that information (Sherwood & Gómez-Robles, 2017). Such markedly distinct evolutionary pressures, if one of them did indeed drive much of human brain evolution, should be readily detectable in modern human psychology.

To test a focused set of hypotheses about human psychology derived from these broad theories, we designed a simple bargaining experiment in which children had the opportunity to use social information in one of two ways, either strategically to exploit an opponent for pay-off advantage or for cultural learning to adapt to a novel circumstance. To incorporate individual experience, we also permitted participants to engage in individual learning by playing the game over four rounds with the same opponent. Our main results show that children's allocations are strongly shaped by cultural learning while showing little strategic use of readily available social information about their partner. As good cultural learners, children in our study may have inferred normative information from the model's distribution strategies. Thus, their behaviour at test may have been more than ‘just’ imitation, reflecting also a developing sensitivity to social norms (House et al., 2019). Both the relationship we observed between a participant's age and making equal allocations and the impact of the demonstrators actions are consistent with prior developmental work on social norm acquisition (House et al., 2013, 2019; Salali et al., 2015).

Complementing this main analysis, we also collected individual-level measures of children's mentalizing skills and their general cognitive abilities. We focused on mentalizing because both the Machiavellian Intelligence Hypothesis and the Cultural Hypothesis point to mentalizing as a key capacity in humans that was probably under autocatalytic selection. Crucially, while the Machiavellian Intelligence Hypothesis predicts that mentalizing skills will be deployed in the service of Machiavellian efforts to strategically out-wit opponents or select partners by anticipating their actions, the Cultural Hypothesis predicts that our greater mentalizing abilities evolved primarily in the service of improving cultural learning. Of course, these two accounts are *not*, broadly speaking, mutually exclusive in making predictions about how and when humans can or are willing to exhibit their capacities for cultural learning or strategic thinking in everyday life across the lifespan. However, in the specific context of our experimental design, the predictions are competing. Straightforwardly, all of our measures of mentalizing predicted superior cultural learning (more selective imitation, less overimitation), but were not reliably associated with using the available social information to predict their partner's behaviour to select pay-off-maximizing options. While this supports the Cultural Hypothesis, it provides no support for the Machiavellian Intelligence Hypothesis with regards to children's behaviour in this bargaining context. Of course, future work may very well reveal the explanatory power of the Machiavellian Intelligence Hypothesis.

In designing this experiment, we strove to ‘tilt’ the situation in favour of Machiavellian thinking in several ways. First, we used a zero-sum social interaction with real pay-offs that we described explicitly to participating children as a ‘game’. Children in this society see ‘games’ as competitive interactions where it is socially approved of to obtain the most points, rewards or, in this case, stickers. We used a zero-sum bargaining game instead of a cooperative game because imitation in the latter can lead to higher pay-offs over repeated interactions. Second, we permitted children to play the same game with the same partner over four rounds. Children might have revealed an initial inclination to copy the demonstrator, but then quickly recognized how their opponent could be exploited. However, they showed little of this type of strategic decision making. Finally, we paired participants with a stranger to avoid any concerns the child might have about interaction after the game. We could have used other children from the daycares, or their teachers, but that would have worked against Machiavellian motivations.

On the other hand, given the evidence suggesting that children tend to copy older and more experienced individuals (e.g. VanderBorght and Jaswal, 2009), one could argue that our setup tilted children towards cultural learning. Although this may indeed be the case, such a finding would confirm another prediction from the Cultural Hypothesis by illustrating the power of model-based learning strategies in a competitive situation with real costs (Laland, 2017; Henrich, 2015a). Future work should vary the age, sex and other characteristics of both the partner and demonstrator.

Another factor that may have influenced our results was the presence of the demonstrator at test. A recent review of the methodological correlates of children's overimitation suggests that, although children do often overimitate when left alone, imitation is more likely when the model that displayed the imitated behaviours remains present at test (Hoehl et al., 2019). However, the social pressure of the model's presence would probably have been constant across our conditions – and thus the differing rates of imitation between the conditions of the sticker game (e.g. near ceiling in the Even condition as compared with Nice/Selfish) require a different explanation. Furthermore, while social pressure may have biased children towards imitation, this does little to explain the reported covariation between mentalizing and rates of imitation of the irrelevant behaviours – better mentalizers were more selective imitators. Thus, there is little reason to suspect that the presence of the demonstrator qualitatively altered these findings, although future work should examine this inference.

In closing, we emphasize our study's limitations. First, there may be other evolutionary hypotheses that we have not considered that could deliver this pattern of results. Second, while much cross-cultural evidence supports the centrality of cultural learning for children, it remains an important concern that we have sampled only a single population (Henrich et al., 2010) and important patterns of variation in children's social behaviour have been observed across societies (House et al., 2013, 2019; Schäfer et al., 2015; Robbins & Rochat, 2011; Henrich & Muthukrishna, 2021). Having refined our protocol and obtained interesting results, we hope to collect similar data in diverse populations. If we are truly seeing a robust product of deep evolutionary forces, we should find qualitatively similar results elsewhere. Finally, here we focused on several measures of mentalizing and one measure of general cognitive ability as a control; future work should collect and explore a larger battery of cognitive measures across a more diverse range of contexts.

## Supplementary material

For supplementary material accompanying this paper visit https://doi.org/10.1017/ehs.2021.11 click here to view supplementary material

## Research transparency and reproducibility

All data and analysis scripts are publicly available on the Open Science Framework: https://osf.io/dgyrj/ (DOI: 10.17605/OSF.IO/DGYRJ)
