# Supplementary material for: Machiavellian strategist or cultural learner? Mentalizing and learning over development in a resource-sharing game
Source: Evol Hum Sci. 2021 Mar 10;3:e14. doi: 10.1017/ehs.2021.11 (PMC10427301; doi:10.1017/ehs.2021.11)
Supplement: Supplementary file 1 [file S2513843X21000116sup001.pdf]

Machiavellian Strategist or Cultural Learner? Mentalizing and learning over development in a resource sharing game

Supplemental Materials

Baimel, Adam*
Department of Psychology, Health and Professional Development
Oxford Brookes University
*abaimel@brookes.ac.uk

Juda, Myriam
Department of Psychology
Simon Fraser University

Birch, Susan
Department of Psychology
University of British Columbia

Henrich, Joseph*
Department of Human Evolutionary Biology
Harvard University
*henrich@fas.harvard.edu

January 29, 2021

S 1 The contributions of mentalizing and cognitive ability to overimitation

S 1.1 False Belief

Table S1: Poisson regression models to predict overimitation from false belief and cognitive ability

	Overimitation				
	Model 1	Model 2	Model 3	Model 4	Model 5
	OR (95% CI)	OR (95% CI)	OR (95% CI)	OR (95% CI)	OR (95% CI)
Intercept	0.438***	0.429***	0.408***	0.494***	0.499***
	(0.271, 0.706)	(0.307, 0.600)	(0.300, 0.556)	(0.372, 0.656)	(0.362, 0.687)
False Belief (1 = Pass)	0.708*	0.583***	0.669**	0.669**	0.652*
	(0.489, 1.026)	(0.389, 0.873)	(0.464, 0.967)	(0.463, 0.966)	(0.416, 1.020)
Cog. Ability (Centered)		1.037**	1.048**	1.048**	1.048**
		(1.006, 1.069)	(1.008, 1.089)	(1.008, 1.089)	(1.010, 1.089)
False Belief X Cog. Ability			0.979	0.979	0.979
			(0.941, 1.019)	(0.941, 1.019)	(0.942, 1.018)
Round (0 = Round 1)				0.874**	0.874**
				(0.774, 0.986)	(0.774, 0.986)
Age (Yrs, Centered)					1.016
					(0.610, 1.690)
Sex (Prop. of Males)					1.223
					(0.727, 2.060)
463 observations					
116 participants					
18 sites					

Notes: Coefficients are presented as odds ratios, so “1” indicates no effect. Standard errors and confidence intervals are robust and use two-way clustering on both individuals and sites. 95% confidence intervals are reported below each coefficient in parentheses. Round of the game was treated as a continuous variable. Sex was centered on the percentage of males to ease interpretation of the other coefficients for the entire sample. For those interested in significance testing, ***, **, and * indicate p-values below 0.01, 0.05 and 0.1.

4 S 1.2 ToM Storybooks

Table S2: Poisson regression models to predict overimitation from ToM storybooks and cognitive ability

	Overimitation				
	Model 1	Model 2	Model 3	Model 4	Model 5
	OR (95% CI)	OR (95% CI)	OR (95% CI)	OR (95% CI)	OR (95% CI)
Intercept	0.332***	0.285***	0.299***	0.379***	0.375***
	(0.234, 0.469)	(0.198, 0.409)	(0.211, 0.423)	(0.256, 0.562)	(0.254, 0.555)
ToM Storybooks (Centered)	0.989	0.974**	0.975*	0.975*	0.967**
	(0.966, 1.012)	(0.950, 0.998)	(0.951, 1.000)	(0.951, 1.000)	(0.942, 0.992)
Cog. Ability (Centered)		1.044***	1.045***	1.045***	1.042***
		(1.017, 1.071)	(1.023, 1.068)	(1.023, 1.068)	(1.022, 1.064)
ToM Storybooks X Cog. Ability			0.999	0.999	0.999
			(0.998, 1.001)	(0.998, 1.001)	(0.998, 1.001)
Round (0 = Round 1)				0.842**	0.842**
				(0.738, 0.961)	(0.738, 0.961)
Age (Years, Centered)					1.396
					(0.858, 2.273)
Sex (Prop. of Males)					1.008
					(0.547, 1.859)
300 observations					
75 participants					
17 sites					

Notes: Coefficients are presented as odds ratios, so “1” indicates no effect. Standard errors and confidence intervals are robust and use two-way clustering on both individuals and sites. 95% confidence intervals are reported below each coefficient in parentheses. Round of the game was treated as a continuous variable. Sex was centered on the percentage of males to ease interpretation of the other coefficients for the entire sample. For those interested in significance testing, ***, **, and * indicate p-values below 0.01, 0.05 and 0.1.

S 1.3 Parental Report - Children's Social Understanding Scale [CSUS]

Table S3: Poisson regression models to predict overimitation from CSUS and cognitive ability

	Overimitation				
	Model 1	Model 2	Model 3	Model 4	Model 5
	OR (95% CI)	OR (95% CI)	OR (95% CI)	OR (95% CI)	OR (95% CI)
Intercept	0.427***	0.333***	0.345***	0.374***	0.368***
	(0.257, 0.711)	(0.215, 0.515)	(0.224, 0.530)	(0.231, 0.604)	(0.223, 0.609)
CSUS (Centered)	0.625	0.389**	0.487	0.487	0.580
	(0.308, 1.266)	(0.163, 0.925)	(0.136, 1.739)	(0.136, 1.743)	(0.137, 2.454)
Cog. Ability (Centered)		1.049***	1.044***	1.044***	1.046***
		(1.021, 1.079)	(1.017, 1.072)	(1.017, 1.072)	(1.017, 1.076)
CSUS X Cog. Ability			0.963	0.963	0.959
			(0.870, 1.067)	(0.870, 1.067)	(0.861, 1.070)
Round (0 = Round 1)				0.947	0.947
				(0.872, 1.029)	(0.872, 1.029)
Age (Years, Centered)					0.849
					(0.495, 1.454)
Sex (Prop. of Males)					1.314
					(0.790, 2.185)
272 observations					
68 participants					
17 sites					

Notes: Coefficients are presented as odds ratios, so "1" indicates no effect. Standard errors and confidence intervals are robust and use two-way clustering on both individuals and sites. 95% confidence intervals are reported below each coefficient in parentheses. Round of the game was treated as a continuous variable. Sex was centered on the percentage of males to ease interpretation of the other coefficients for the entire sample. For those interested in significance testing, ***, **, and * indicate p-values below 0.01, 0.05 and 0.1.

S 1.4 Cognitive Ability Subscale Analysis

We modeled whether the relationships between cognitive ability and overimitation presented in the main text (see Table 3) could be further qualified by examining the associations between the three subscales of the Brief Intellectual Ability [BIA] test and amount of overimitation. The BIA score is made up from the equally-weighted results of three individual tests - a test of (1) concept formation, (2) verbal comprehension, and (3) visual matching. The concept formation test asks participants to identify rules that define patterns in sequences of geometric figures. The verbal comprehension test asks participants to name pictured objects, identify synonyms and antonyms of said word. The visual matching test has participants identify (e.g., point to) as many of matching pairs of numbers in a row of six numbers as quickly as they can in a three-minute time period. The subcsale analyses reveal that the already small effect of cognitive ability on overimitation may be driven mostly by verbal and visual matching scores rather than concept formation (see Table S4 for details).

Table S4: Poisson regression models to predict overimitation by mentalizing and the subscales of the cognitive ability test

	Overimitation		
	False Belief	ToM Storybooks	CSUS
	OR (95% CI)	OR (95% CI)	OR (95% CI)
Intercept	0.448***	0.280***	0.342***
	(0.314, 0.640)	(0.197, 0.398)	(0.203, 0.577)
Mentalizing	0.578***	0.977*	0.378**
	(0.394, 0.847)	(0.953, 1.001)	(0.175, 0.819)
BIA - Concept Formation (Centered)	1.001	1.013	0.984
	(0.978, 1.025)	(0.995, 1.031)	(0.950, 1.019)
BIA - Verbal (Centered)	1.016**	1.015*	1.022***
	(1.002, 1.030)	(0.999, 1.031)	(1.008, 1.037)
BIA - Visual Matching (Centered)	1.015	1.027**	1.037**
	(0.979, 1.052)	(1.000, 1.055)	(1.008, 1.067)
Observations	447	292	256
Participants	112	73	64
Sites	18	17	16

Notes: Coefficients are presented as odds ratios, so "1" indicates no effect. Standard errors and confidence intervals are robust and use two-way clustering on both individuals and sites. 95% confidence intervals are reported below each coefficient in parentheses. For those interested in significance testing, ***, **, and * indicate p-values below 0.01, 0.05 and 0.1.

S 2 The contributions of mentalizing and cognitive ability to payoff maximizing decisions

S 2.1 False Belief

Table S5: Logistic regression models to predict payoff maximizing decisions from false belief and cognitive ability

	Sticker allocations (1 = Payoff maximizing choice)			
	Model 1	Model 2	Model 3	Model 4
	OR (95% CI)	OR (95% CI)	OR (95% CI)	OR (95% CI)
Intercept	2.486***	15.735***	12.393***	14.302***
	(1.368, 4.517)	(4.850, 51.053)	(3.253, 47.222)	(4.044, 50.577)
False Belief (1 = Pass)	0.845	0.729	0.726	0.592
	(0.422, 1.690)	(0.312, 1.703)	(0.308, 1.709)	(0.240, 1.461)
Cog. Ability (Centered)	1.005	1.003	1.003	1.000
	(0.977, 1.033)	(0.976, 1.031)	(0.976, 1.031)	(0.972, 1.030)
Even Condition		0.511	0.510	0.483
		(0.140, 1.860)	(0.140, 1.860)	(0.150, 1.558)
Nice Condition		0.216**	0.215**	0.210***
		(0.061, 0.766)	(0.061, 0.758)	(0.067, 0.662)
Selfish Condition		0.047***	0.046***	0.046***
		(0.015, 0.143)	(0.015, 0.138)	(0.018, 0.118)
Round (0 = Round 1)			1.187	1.189
			(0.964, 1.460)	(0.964, 1.467)
Age (Yrs. Centered)				1.495
				(0.831, 2.690)
Sex (0 = Prop. of Males)				0.910
				(0.452, 1.830)
Observations = 463				
Participants = 116				
Sites = 18				

Notes: Coefficients are presented as odds ratios, so "1" indicates no effect. Standard errors and confidence intervals are robust and use two-way clustering on both individuals and sites. 95% confidence intervals are reported below each coefficient in parentheses. The CONTROL condition (Intercept; controlling for other variables) is the reference category for condition effects. Round of the game was treated as a continuous variable. Sex was centered on the percentage of males to ease interpretation of the other coefficients for the entire sample. For those interested in significance testing, ***, **, and * indicate p-values below 0.01, 0.05 and 0.1.

S 2.2 ToM Storybooks

Table S6: Logistic regression models to predict payoff maximizing decisions from ToM storybooks and cognitive ability

	Sticker allocations (1 = Payoff maximizing choice)			
	Model 1	Model 2	Model 3	Model 4
	OR (95% CI)	OR (95% CI)	OR (95% CI)	OR (95% CI)
Intercept	2.472***	14.616***	11.122***	11.336***
	(1.583, 3.860)	(4.107, 52.011)	(2.401, 51.514)	(2.260, 56.852)
ToM Storybooks (Centered)	1.015	1.012	1.013	1.018
	(0.989, 1.042)	(0.984, 1.042)	(0.983, 1.043)	(0.982, 1.056)
Cog. Ability (Centered)	1.007	1.004	1.004	1.001
	(0.969, 1.047)	(0.964, 1.046)	(0.964, 1.046)	(0.953, 1.051)
Even Condition		0.560	0.559	0.564
		(0.113, 2.788)	(0.112, 2.792)	(0.113, 2.820)
Nice Condition		0.153**	0.151**	0.142**
		(0.030, 0.786)	(0.030, 0.768)	(0.025, 0.796)
Selfish Condition		0.051***	0.050***	0.050***
		(0.012, 0.210)	(0.012, 0.201)	(0.011, 0.223)
Round (0 = Round 1)			1.217	1.217
			(0.922, 1.606)	(0.920, 1.610)
Age (Yrs. Centered)				0.870
				(0.379, 1.995)
Sex (0 = Prop. of Males)				0.734
				(0.353, 1.527)
Observations = 299				
Participants = 75				
Sites = 17				

Notes: Coefficients are presented as odds ratios, so "1" indicates no effect. Standard errors and confidence intervals are robust and use two-way clustering on both individuals and sites. 95% confidence intervals are reported below each coefficient in parentheses. The CONTROL condition (Intercept; controlling for other variables) is the reference category for condition effects. Round of the game was treated as a continuous variable. Sex was centered on the percentage of males to ease interpretation of the other coefficients for the entire sample. For those interested in significance testing, ***, **, and * indicate p-values below 0.01, 0.05 and 0.1.

S 2.3 Parental Report [CSUS]

Table S7: Logistic regression models to predict payoff maximizing decisions from CSUS and cognitive ability

	Sticker allocations (1 = Payoff maximizing choice)			
	Model 1	Model 2	Model 3	Model 4
	OR (95% CI)	OR (95% CI)	OR (95% CI)	OR (95% CI)
Intercept	1.891***	9.172***	6.859***	7.339***
	(1.229, 2.909)	(3.528, 23.845)	(2.070, 22.731)	(2.332, 23.096)
CSUS (Centered)	0.646	0.601	0.595	0.301
	(0.159, 2.618)	(0.124, 2.902)	(0.121, 2.925)	(0.053, 1.699)
Cog. Ability (Centered)	1.006	1.007	1.007	1.002
	(0.968, 1.046)	(0.972, 1.044)	(0.971, 1.045)	(0.965, 1.039)
Even Condition		0.462	0.459	0.403
		(0.146, 1.463)	(0.144, 1.462)	(0.117, 1.394)
Nice Condition		0.293*	0.290*	0.263*
		(0.076, 1.134)	(0.075, 1.120)	(0.066, 1.057)
Selfish Condition		0.057***	0.055***	0.057***
		(0.020, 0.163)	(0.020, 0.154)	(0.021, 0.157)
Round (0 = Round 1)			1.232	1.244
			(0.952, 1.593)	(0.953, 1.623)
Age (Yrs. Centered)				2.165*
				(0.926, 5.062)
Sex (0 = Prop. of Males)				0.705
				(0.244, 2.038)
Observations = 271				
Participants = 68				
Sites = 17				

Notes: Coefficients are presented as odds ratios, so "1" indicates no effect. Standard errors and confidence intervals are robust and use two-way clustering on both individuals and sites. 95% confidence intervals are reported below each coefficient in parentheses. The CONTROL condition (Intercept; controlling for other variables) is the reference category for condition effects. Round of the game was treated as a continuous variable. Sex was centered on the percentage of males to ease interpretation of the other coefficients for the entire sample. For those interested in significance testing, ***, **, and * indicate p-values below 0.01, 0.05 and 0.1.

S 3 Supplemental Study: Exploring children's comprehension of the sticker game

In our main study, children only ever saw the demonstrator perform one particular allocation and this had a big impact on children's own allocations. An important question is what children inferred from this demonstration. Since our instructions implied that participants could allocate the stickers however they wanted, children most likely inferred that the model's action represented either a 'good strategy' in this interaction or the normatively correct standard in this situation. Either inference is consistent with view assumed in the main text. However, children may have inferred from the model's demonstration that the only permissible action was to allocate the stickers in precisely the same manner as the model. The view

is subtly but importantly different from inferring something normative. As an analogy, young basketball players might watch an experienced player shoot using an underhand technique (e.g. NBA star Rick Barry). They might assume that you must shoot underhanded in basketball (or else it doesn't count and causes a 'turnover'); or, they might see this as the usual approach that people take in shooting, but that you can shoot overhand if you prefer (but others may think it is a bit odd). To examine this question, we conducted a small supplemental study in which children played the sticker game in an identical manner as in our main study. Following the game, children were asked a series of questions regarding the interaction to determine how children understood the 'rules' of the game.

S 3.1 Methods

Forty-four children were recruited from the Living Lab at The Telus World of Science Museum in Vancouver, Canada. Five of these participants were excluded from all analyses for three reasons (1) experimenter error (incorrect instructions were given to the child during the observation phase), (2) difficulties with answering the comprehension check questions in English or (3) having watched a sibling play the sticker game prior to participating. Our final sample of 39 contained 17 females and ranged in age from 3.58 to 6.93 years (M = 5.22, SD = 1.07).

Participants in this study were randomly assigned to one of two conditions (EVEN: N = 20; SELFISH: N = 19. The CONTROL condition from Study 1 was not replicated here, as there was no cause for concern regarding imitation effects as allocations were occluded from the participants' view. We included the SELFISH condition (but not the NICE condition) because if responses to follow-up questions in the SELFISH condition indicate that children understood that the stickers could be distributed differently than how they had observed, yet continued to imitate the unfavorable uneven distribution that resulted in reduced sticker payoffs, we could be more confident that these behaviors are the result of a propensity for imitation and not a lack of understanding or strict rule following. All participants played the game with the same two female experimenters who played the same role (proposer or responder) with each participant. Otherwise, the sticker game proceeded exactly as described in the main study. After the game, the experimenter who had played as proposer in the sticker game asked the participant six questions. These questions are described in tandem with the results below.

S 3.2 Results

In this section, we first show that we replicated the relevant results from the main text in this supplemental experiment and then explore how our participants understood the game using our interview protocol.

S 3.2.1 Replicating relevant results

As in the main study, children's allocations were strongly influenced by the allocation strategy they saw in the observation phase (see Figure S1 and Table S8 for model summary details). Note that the regression coefficients here, expressed in odds ratios, are relative to the SELFISH condition, (not a CONTROL condition as is presented in the main text), which is why they are so large. The confidence intervals are large because with 80 total observations in EVEN Condition, we have only 5 *uneven* observations. Nevertheless, the main results for these conditions in the main text are replicated here.

Table S8: Logistic regression models to predict uneven/even allocations in Study 2

	Sticker Allocations (0 = Uneven; 1 = Even)			
	Model 1	Model 2	Model 3	Model 4
	(1)	(2)	(3)	(4)
Intercept	1.303	0.910	0.930	0.878
	(0.614, 2.764)	(0.400, 2.071)	(0.383, 2.258)	(0.360, 2.139)
Even Condition	11.512^{***}	11.828^{***}	11.444^{**}	14.943^{***}
	(1.816, 72.963)	(1.810, 77.308)	(1.386, 94.501)	(2.012, 110.981)
Round (0 = Round 1)		1.274^{*}	1.275^{*}	1.328^{*}
		(0.999, 1.625)	(0.998, 1.629)	(0.987, 1.785)
Age (Yrs. Centered)			1.121	1.121
			(0.402, 3.123)	(0.399, 3.147)
Sex (0 = Prop. of Males)			1.105	1.106
			(0.222, 5.514)	(0.219, 5.586)
Even Condition X Round				0.821
				(0.576, 1.168)
Observations = 156				
Participants = 39				

Notes: Coefficients are presented as odds ratios, so "1" indicates no effect. Standard errors and confidence intervals are robust and clustered on individuals. 95% confidence intervals are reported below each coefficient in parentheses. The SELFISH condition (Intercept; controlling for other variables) is the reference category for condition effects. Round of the game was treated as a continuous variable. Sex was centered on the percentage of males to ease interpretation of the other coefficients for the entire sample. For those interested in significance testing, ***, **, and * indicate p-values below 0.01, 0.05 and 0.1.

S 3.2.2 Participant's comprehension of the game

The post-game interviews of these participants unfolded as follows. First, at the completion of game, the experimenter exclaimed that the other research assistant had forgotten the rules of the game, and asked whether or not the child could teach her how to play the game. The child was then asked to indicate whether not the experimenter was allowed to distribute stickers in (1) an even manner (two in each basket), (2) uneven manner (three in one basket and one in the other), and (3) another uneven manner in which four stickers were placed in one basket and none in the other. Overall, across both conditions, roughly 60% of participants explicitly expressed the view that they could have done something different from the demonstrator and only 1 participant out of 39 said that an even distribution was not acceptable. This implies that participants didn't see deviations form the allocations they observed as rule violations.

However, children's inferences about the situation were not symmetrical across our two conditions. Crucially, participants in the SELFISH condition saw it as permissible to payoff maximize by making even offers; but, despite this recognition, they tended to copy the allocations of their demonstrator. Yet, in the EVEN condition, a small majority of participants (12 out of 20) thought that an uneven distribution would *not* be allowed. We cannot be sure whether children felt an uneven distribution was non-normative or an actual rule violation. This also means that 30% of participants thought that uneven distributions were permissible.

Participants were then asked if they remembered what the proposer in the observation phase did on her turns in the game and to indicate how many stickers she had put in each basket. Six children in the SELFISH condition and 7 children in the EVEN condition said they did not remember the allocations. Of those who did recall, 1 child out of 11 incorrectly stated the demonstrator's allocation in the EVEN condition

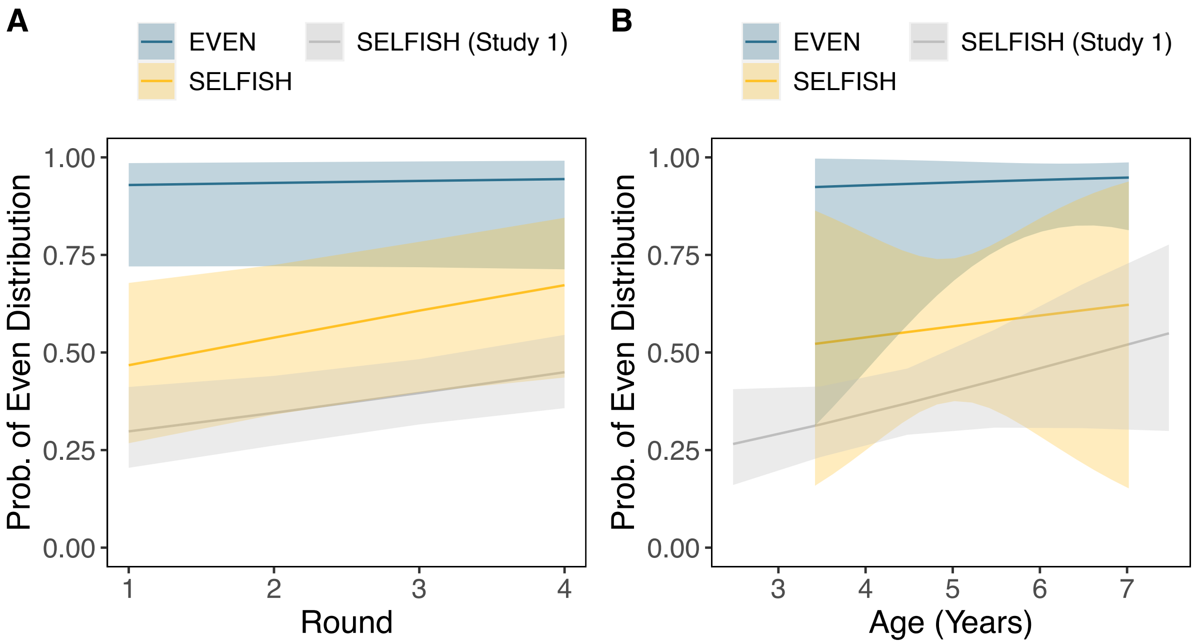

Figure S1: **Predicted probability of even distributions in the two conditions of Study 2 across the four rounds (Panel A) and age (Panel B)**. Predictions were generated from Model 4 in S8. The shaded regions show the 95% confidence intervals based on subject-level clustering. The grey lines reproduce predicted estimates from the SELFISH condition in Study 1 for comparison. Study 1 recruited children of a wider age-range than Study 2.

as did 2 out of 13 in the SELFISH condition. Then, children's memory of their own behaviors in the game was assessed in the same manner. Six children incorrectly remembered their own decisions: 2 in the EVEN condition and 4 in the SELFISH condition.

Following these memory checks, the experimenter recounted how the model distributed stickers in the observation phase and what the child did at test and then asked, "Could you have put the stickers in the baskets in any other way?". The results were almost identical with those above. Again, nearly two-third of participants explained that they could have deviated from the demonstrators' allocation. However, in the EVEN condition, 12 out of 20 children again thought that an uneven distribution would not be allowed. In the SELFISH condition, 2 children out of 19 thought only the demonstrators uneven allocation was allowed–that is, 17 children thought they could deviate from what they saw the demonstrator do.

Next, participants were asked, "Would you have been allowed to just take the stickers without even putting them into the baskets?" The answer to which is technically 'yes', however we wanted to see if children understood this situation to be a game with a certain set of boundary conditions. And unlike the other questions we asked, this question provided a response in which the expected modal answer would be 'no'. Indeed, only 8 participants (3 in the EVEN condition, and 5 in the SELFISH condition) said that they could have taken the stickers without first putting them in the baskets.

Lastly, we probed whether participants could explicitly reason about sticker distribution strategies by asking them, "While you were playing, if you thought [name of experimenter] was always going to choose the basket with the most stickers in it, how would you play the game in order to get the most stickers?" This was an open-ended question and responses were later coded for the presence/absence of mentioning an even distribution which is the strategic allocation given uncertainty regarding the responder's decisions in the EVEN condition, and knowing that the responder was SELFISH in the other condition. Many children provided no or irrelevant answers. Of those that did provide a relevant answer (11 in the EVEN condition

and 12 in the SELFISH condition); 9 in the EVEN condition hinted at an explicit understanding that an even distribution was the best strategy, where as only 4 explicitly reported the same in the SELFISH condition.

S 3.2.3 Discussion

In this supplemental study, we sought to replicate certain key results from the main text and to probe children's explicit understanding of the rules of the sticker game. Despite the small sample size, the results from the main text replicate. On the question of children's inferences about normativity or permissibility of certain allocations in the game, we find a nuanced picture. Crucially, in the SELFISH condition where copying the model's allocations results in the participant getting fewer stickers, children overwhelmingly felt that they could deviate from the model's allocations, either by allocating 2/2 or 4/0 stickers. This means that the costly allocations of participants in the SELFISH treatment cannot be explained by confusion about the rules. This relieves an important methodological concern as it shows that our instructions themselves didn't lead children to automatically infer that they had to do whatever their demonstrator did.

However, we did find an interaction of the condition with our instructions. The impact of the demonstrators actions in the EVEN condition seemed to steer a small majority of participants toward the view that only the even allocation would be permitted. Here, the cultural transmitted information, perhaps because it dovetailed with some expectations that children brought into the lab with them about equal splits, caused some to infer that only an even split was permitted. Notably, older children were more likely to say that 2/2 was the only allocation permitted (Saying that alternative allocations were allowed was negatively correlated with age: r = -.64 for "3/1" allocations and r = -.76 for "4/0" allocations in the EVEN condition). Of course, some 40% of participants in the EVEN condition didn't make that inference. These data suggest how cultural learning shapes people's construction of the"rules of the game" and is likely relevant to understanding institutions. This finding underlines the centrality of cultural learning in children and certainly isn't the kind of mistaken inference that we'd expect under the Machiavellian Intelligence Hypothesis.

Note, although we find these results sufficient to relieve our concerns that our instructions may been misleading to children across the board, we haven't included this speculative discussion in the main text given the sample sizes and uncertainties involved.
